# 5-Diethyl­amino-2-[(*E*)-(2,4-dimeth­oxy­phen­yl)imino­meth­yl]phenol

**DOI:** 10.1107/S160053681201882X

**Published:** 2012-05-02

**Authors:** Esen Nur Kantar, Yavuz Köysal, Sümeyye Gümüş, Erbil Ağar, Mustafa Serkan Soylu

**Affiliations:** a Department of Physics, Faculty of Arts and Sciences, Ondokuz Mayıs University, TR-55139 Samsun, Turkey; b Yesilyurt Demir Celik Vocational School, Ondokuz Mayıs University, TR-55139 Samsun, Turkey; c Department of Chemistry, Faculty of Arts and Sciences, Ondokuz Mayıs University, Kurupelit, 55139 Samsun, Turkey; d Department of Physics, Faculty of Arts and Sciences, Giresun University, Giresun, Turkey

## Abstract

The title Schiff base, C_19_H_24_N_2_O_3_, exists in the crystal structure in the phenol–imine tautomeric form with an intra­molecular O—H⋯N hydrogen bond. The planes of the aromatic rings form a dihedral angle of 36.8 (8)°. The crystal packing is characterized by C—H⋯O hydrogen bonds and π–π stacking inter­actions [centroid–centroid distance = 3.478 (4)Å].

## Related literature

Schiff bases of salicyl­aldehyde may exhibit thermochromism or photochromism, depending on the planarity or non-planarity, respectively, of the mol­ecule, see: Amimoto & Kawato (2005 ▶); Schmidt & Cohen (1964 ▶). For similar structures, see: Ha (2011 ▶); Asiri *et al.* (2010 ▶). For hydrogen-bond motifs, see: Bernstein *et al.* (1995 ▶).
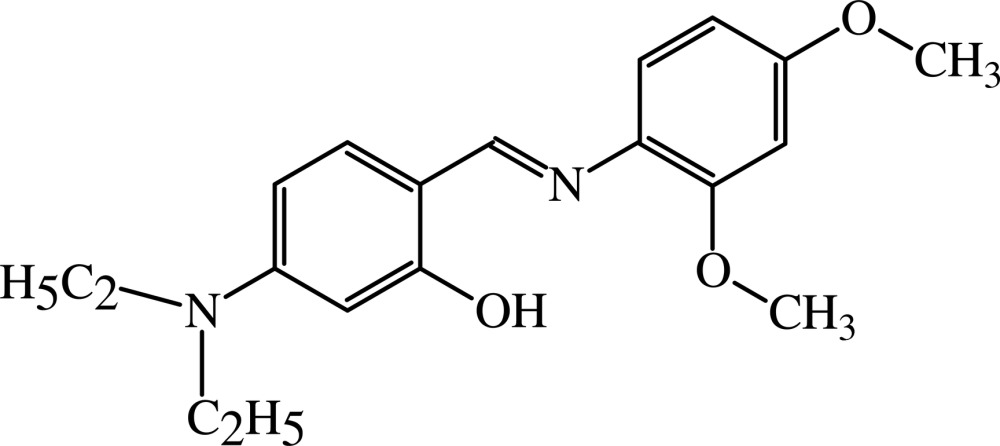


## Experimental

### 

#### Crystal data

C_19_H_24_N_2_O_3_
*M* *_r_* = 328.40
Monoclinic, 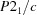

*a* = 7.2028 (3) Å
*b* = 9.4423 (5) Å
*c* = 26.050 (2) Å
β = 91.742 (7)°
*V* = 1770.9 (2) Å^3^
*Z* = 4
Mo *K*α radiation
μ = 0.08 mm^−1^
*T* = 293 K
0.22 × 0.15 × 0.10 mm

#### Data collection

Oxford Diffraction SuperNova (single source at offset) Eos diffractometer
Absorption correction: multi-scan (*CrysAlis PRO*; Oxford Diffraction, 2007 ▶) *T*_min_ = 0.933, *T*_max_ = 1.000
8031 measured reflections
4165 independent reflections
1959 reflections with *I* > 2σ(*I*)
*R*_int_ = 0.036

#### Refinement

*R*[*F* ^2^ > 2σ(*F* ^2^)] = 0.064
*wR*(*F* ^2^) = 0.164
*S* = 1.06
4165 reflections
226 parameters
1 restraint
H atoms treated by a mixture of independent and constrained refinement
Δρ_max_ = 0.16 e Å^−3^
Δρ_min_ = −0.15 e Å^−3^

### 

Data collection: *CrysAlis PRO* (Oxford Diffraction, 2007 ▶); cell refinement: *CrysAlis PRO*; data reduction: *CrysAlis PRO*; program(s) used to solve structure: *SHELXS97* (Sheldrick, 2008 ▶); program(s) used to refine structure: *SHELXL97* (Sheldrick, 2008 ▶); molecular graphics: *ORTEP-3 for Windows* (Farrugia, 1997 ▶); software used to prepare material for publication: *WinGX* (Farrugia, 1999 ▶).

## Supplementary Material

Crystal structure: contains datablock(s) I, global. DOI: 10.1107/S160053681201882X/ld2055sup1.cif

Structure factors: contains datablock(s) I. DOI: 10.1107/S160053681201882X/ld2055Isup2.hkl

Supplementary material file. DOI: 10.1107/S160053681201882X/ld2055Isup3.cml

Additional supplementary materials:  crystallographic information; 3D view; checkCIF report

## Figures and Tables

**Table 1:** Hydrogen-bond geometry (Å, °)

*D*—H⋯*A*	*D*—H	H⋯*A*	*D*⋯*A*	*D*—H⋯*A*
O1—H1⋯N1	0.84 (2)	1.79 (2)	2.575 (3)	153 (4)
C16—H16*B*⋯O1^i^	0.97	2.53	3.491 (4)	172

Symmetry code: (i) 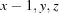.

**Fig. 1.**
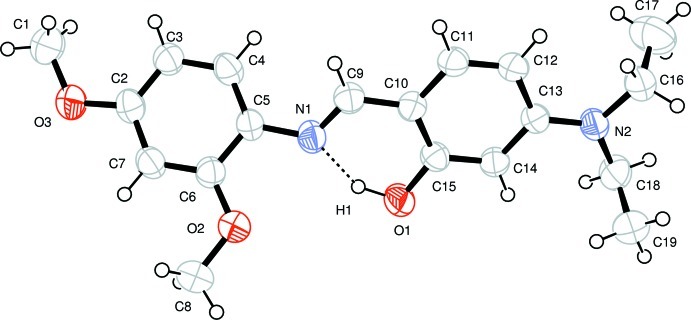
A view of (I) with 50% probability displacement ellipsoids.

## supplementary crystallographic information

### Comment

We have studied a Schiff base derived from 4-(diethylamino)-2-hydroxybenzaldehyde. It is known that Schiff bases of salicylaldehyde may exhibit thermochromism or photochromism, depending on planarity or non-planarity of the molecule, respectively (Schmidt & Cohen, 1964; Amimoto & Kawato, 2005).

The C10-C9-N1-C5 torsion angle is -169.8 (2)°, that contributes to the general non-planarity of the molecule. The C15-O1 [1.352 (3)Å] bond length is similar to the corresponding distance in 4-Bromo-2- {[(pyridin-3-ylmethyl)imino]-methyl}phenol, [1.352 (4)Å; Ha, 2011] and in the monoclinic modification of 2-[(1,3-benzothiazol-2-yl)iminomethyl]phenol [1.345 (3)Å; Asiri *et al.*, 2010).

Depending on the tautomers, two types of intramolecular hydrogen bonds are observed in Schiff bases: O-H···N in phenol-imine and N-H···O in keto-amine tautomers. Our X-ray investigation shows that the title compound exists in the phenol-imine form. The title compound forms intermolecular C-H···O and a strong intramolecular O-H···N hydrogen bonds, namely C16-H16B···O1 [symmetry code: (i) x-1,y,z] and O1-H1A···N1. The intramolecular hydrogen bonds generates a six membered ring, producing S(6) ring motif (Bernstein, *et al.*, 1995). Weak π-π stacking interactions are observed which may influence crystal stability – the distance between centroids Cg1(C2-C7 ring) to Cg1^ii^ [symmetry code: (ii) 1-x,-y,1-z] is 3.478 (4)Å.

### Experimental

The 2-{(*E*)-[(2,4-dimethoxyphenyl)imino]methyl}-5- (pentan-3-yl)phenol was prepared by refluxing a mixture of 4-(diethylamino)-2-hydroxybenzaldehyde (0.011 g 0.057 mmol) in 20 ml of ethanol and 2,4-dimethoxyaniline (0.009 g 0.057 mmol) in 20 ml of ethanol. The mixture was stirred for 1 h under reflux. The crystals of the 2-{(*E*)-[(2,4-dimethoxyphenyl)imino]methyl}-5-(pentan-3-yl)phenol suitable for X-ray analysis were obtained from ethyl alcohol by slow evaporation (yield %72; m.p. 371–373 °K).

### Refinement

The structure was solved by direct methods and refined by full-matrix least-square techniques. All H atoms were located geometrically and refined using a riding model, except for atom H1 bonded to atom O1, which was freely refined. The C—H distances were fixed at 0.93-0.97 Å. The hydrogen atoms of methyl groups were placed in a staggered conformation.

### Crystal data

**Table:**

C_19_H_24_N_2_O_3_	*F*(000) = 704
*M**_r_* = 328.40	*D*_x_ = 1.232 Mg m^−^^3^
Monoclinic, *P*2_1_/*c*	Mo *K*α radiation, λ = 0.71073 Å
*a* = 7.2028 (3) Å	Cell parameters from 1690 reflections
*b* = 9.4423 (5) Å	θ = 3.2–29.0°
*c* = 26.050 (2) Å	µ = 0.08 mm^−^^1^
β = 91.742 (7)°	*T* = 293 K
*V* = 1770.9 (2) Å^3^	Block, orange
*Z* = 4	0.22 × 0.15 × 0.10 mm

### Data collection

**Table:**

Oxford Diffraction SuperNova (single source at offset) Eos diffractometer	4165 independent reflections
Radiation source: SuperNova (Mo) X-ray Source	1959 reflections with *I* > 2σ(*I*)
Mirror monochromator	*R*_int_ = 0.036
Detector resolution: 16.0454 pixels mm^-1^	θ_max_ = 29.1°, θ_min_ = 3.2°
ω scans	*h* = −7→9
Absorption correction: multi-scan (*CrysAlis PRO*; Oxford Diffraction, 2007)	*k* = −9→11
*T*_min_ = 0.933, *T*_max_ = 1.000	*l* = −35→33
8031 measured reflections	

### Refinement

**Table:**

Refinement on *F*^2^	Secondary atom site location: difference Fourier map
Least-squares matrix: full	Hydrogen site location: inferred from neighbouring sites
*R*[*F*^2^ > 2σ(*F*^2^)] = 0.064	H atoms treated by a mixture of independent and constrained refinement
*wR*(*F*^2^) = 0.164	*w* = 1/[σ^2^(*F*_o_^2^) + (0.0373*P*)^2^ + 0.4437*P*] where *P* = (*F*_o_^2^ + 2*F*_c_^2^)/3
*S* = 1.06	(Δ/σ)_max_ < 0.001
4165 reflections	Δρ_max_ = 0.16 e Å^−^^3^
226 parameters	Δρ_min_ = −0.15 e Å^−^^3^
1 restraint	Extinction correction: *SHELXL97* (Sheldrick, 2008), Fc^*^=kFc[1+0.001xFc^2^λ^3^/sin(2θ)]^-1/4^
Primary atom site location: structure-invariant direct methods	Extinction coefficient: 0.0031 (6)

### Special details

**Table:**

Experimental. Absorption correction: CrysAlisPro, Agilent Technologies, Version 1.171.35.19 Empirical absorption correction using spherical harmonics, implemented in SCALE3 ABSPACK scaling algorithm.
Geometry. All e.s.d.'s (except the e.s.d. in the dihedral angle between two l.s. planes) are estimated using the full covariance matrix. The cell e.s.d.'s are taken into account individually in the estimation of e.s.d.'s in distances, angles and torsion angles; correlations between e.s.d.'s in cell parameters are only used when they are defined by crystal symmetry. An approximate (isotropic) treatment of cell e.s.d.'s is used for estimating e.s.d.'s involving l.s. planes.
Refinement. Refinement of *F*^2^ against ALL reflections. The weighted *R*-factor *wR* and goodness of fit *S* are based on *F*^2^, conventional *R*-factors *R* are based on *F*, with *F* set to zero for negative *F*^2^. The threshold expression of *F*^2^ > σ(*F*^2^) is used only for calculating *R*-factors(gt) *etc*. and is not relevant to the choice of reflections for refinement. *R*-factors based on *F*^2^ are statistically about twice as large as those based on *F*, and *R*- factors based on ALL data will be even larger.

### Fractional atomic coordinates and isotropic or equivalent isotropic displacement parameters (Å^2^)

**Table:**

	*x*	*y*	*z*	*U*_iso_*/*U*_eq_	
O1	0.3547 (3)	0.5391 (2)	0.37257 (9)	0.0640 (6)	
N1	0.3519 (3)	0.2811 (2)	0.40463 (9)	0.0529 (6)	
O3	0.7902 (3)	−0.1820 (2)	0.46150 (9)	0.0732 (7)	
C13	−0.1045 (4)	0.6216 (3)	0.31791 (10)	0.0455 (6)	
C14	0.0834 (3)	0.6313 (3)	0.33320 (10)	0.0490 (7)	
H14	0.1488	0.7136	0.3260	0.059*	
O2	0.6402 (3)	0.3076 (2)	0.47090 (8)	0.0650 (6)	
C12	−0.1986 (4)	0.4935 (3)	0.32942 (11)	0.0521 (7)	
H12	−0.3229	0.4830	0.3195	0.062*	
C10	0.0799 (4)	0.3947 (3)	0.37000 (10)	0.0458 (6)	
N2	−0.1934 (3)	0.7311 (2)	0.29284 (9)	0.0555 (6)	
C15	0.1737 (4)	0.5215 (3)	0.35868 (10)	0.0469 (7)	
C11	−0.1092 (4)	0.3860 (3)	0.35480 (10)	0.0502 (7)	
H11	−0.1751	0.3043	0.3623	0.060*	
C9	0.1748 (4)	0.2768 (3)	0.39315 (10)	0.0505 (7)	
H9	0.1083	0.1950	0.4002	0.061*	
C7	0.7176 (4)	0.0578 (3)	0.46522 (11)	0.0552 (8)	
H7	0.8223	0.0689	0.4866	0.066*	
C5	0.4521 (4)	0.1580 (3)	0.41991 (10)	0.0491 (7)	
C16	−0.3951 (4)	0.7321 (3)	0.28369 (12)	0.0628 (8)	
H16A	−0.4404	0.8283	0.2871	0.075*	
H16B	−0.4527	0.6749	0.3097	0.075*	
C2	0.6713 (4)	−0.0755 (3)	0.44630 (11)	0.0550 (7)	
C4	0.4107 (4)	0.0244 (3)	0.40130 (11)	0.0559 (8)	
H4	0.3081	0.0130	0.3791	0.067*	
C6	0.6091 (4)	0.1742 (3)	0.45242 (10)	0.0505 (7)	
C3	0.5170 (4)	−0.0937 (3)	0.41452 (11)	0.0566 (8)	
H3	0.4846	−0.1830	0.4022	0.068*	
C18	−0.0953 (4)	0.8577 (3)	0.27682 (12)	0.0652 (9)	
H18A	−0.1594	0.8975	0.2469	0.078*	
H18B	0.0288	0.8314	0.2669	0.078*	
C8	0.7964 (4)	0.3284 (3)	0.50488 (13)	0.0752 (10)	
H8A	0.9086	0.3126	0.4867	0.113*	
H8B	0.7905	0.2631	0.5330	0.113*	
H8C	0.7954	0.4236	0.5178	0.113*	
C17	−0.4527 (5)	0.6764 (4)	0.23138 (14)	0.1018 (13)	
H17A	−0.3987	0.7341	0.2054	0.153*	
H17B	−0.5856	0.6790	0.2274	0.153*	
H17C	−0.4103	0.5805	0.2280	0.153*	
C19	−0.0808 (5)	0.9695 (3)	0.31823 (14)	0.0801 (11)	
H19A	−0.0146	1.0500	0.3056	0.120*	
H19B	−0.0154	0.9316	0.3478	0.120*	
H19C	−0.2031	0.9981	0.3276	0.120*	
C1	0.7411 (5)	−0.3221 (3)	0.44663 (14)	0.0798 (11)	
H1A	0.7206	−0.3257	0.4101	0.120*	
H1B	0.6296	−0.3496	0.4633	0.120*	
H1C	0.8399	−0.3857	0.4565	0.120*	
H1	0.388 (5)	0.462 (3)	0.3860 (14)	0.114 (15)*	

### Atomic displacement parameters (Å^2^)

**Table:**

	*U*^11^	*U*^22^	*U*^33^	*U*^12^	*U*^13^	*U*^23^
O1	0.0442 (12)	0.0605 (14)	0.0867 (17)	−0.0032 (10)	−0.0064 (11)	0.0111 (12)
N1	0.0546 (14)	0.0542 (15)	0.0494 (14)	0.0062 (11)	−0.0060 (12)	0.0042 (11)
O3	0.0689 (14)	0.0558 (13)	0.0934 (17)	0.0147 (11)	−0.0204 (13)	−0.0056 (11)
C13	0.0443 (15)	0.0474 (16)	0.0449 (16)	0.0009 (12)	0.0014 (13)	0.0003 (12)
C14	0.0436 (15)	0.0456 (16)	0.0580 (17)	−0.0044 (12)	0.0028 (13)	0.0070 (13)
O2	0.0654 (13)	0.0550 (13)	0.0735 (15)	0.0015 (10)	−0.0157 (12)	−0.0042 (10)
C12	0.0437 (15)	0.0492 (17)	0.0631 (19)	−0.0033 (13)	−0.0024 (14)	−0.0031 (14)
C10	0.0467 (15)	0.0437 (15)	0.0470 (16)	0.0012 (12)	0.0017 (13)	0.0017 (12)
N2	0.0472 (13)	0.0534 (15)	0.0654 (16)	−0.0001 (11)	−0.0068 (12)	0.0108 (12)
C15	0.0406 (15)	0.0517 (17)	0.0486 (16)	−0.0010 (13)	0.0019 (12)	0.0009 (13)
C11	0.0457 (15)	0.0483 (16)	0.0568 (18)	−0.0046 (13)	0.0029 (14)	0.0003 (13)
C9	0.0490 (16)	0.0515 (17)	0.0509 (17)	−0.0007 (13)	0.0031 (14)	0.0018 (13)
C7	0.0530 (17)	0.0609 (19)	0.0510 (18)	0.0062 (15)	−0.0087 (14)	−0.0018 (14)
C5	0.0474 (16)	0.0502 (17)	0.0493 (17)	0.0055 (13)	−0.0031 (13)	0.0036 (13)
C16	0.0574 (19)	0.0621 (19)	0.068 (2)	0.0029 (15)	−0.0073 (16)	0.0068 (16)
C2	0.0499 (17)	0.0593 (19)	0.0557 (18)	0.0098 (14)	−0.0001 (14)	0.0012 (14)
C4	0.0554 (18)	0.0647 (19)	0.0470 (17)	0.0034 (15)	−0.0088 (14)	0.0001 (14)
C6	0.0524 (16)	0.0502 (17)	0.0487 (17)	0.0023 (14)	−0.0002 (14)	0.0012 (13)
C3	0.0610 (18)	0.0526 (17)	0.0556 (18)	0.0064 (15)	−0.0049 (15)	−0.0051 (14)
C18	0.065 (2)	0.0584 (19)	0.072 (2)	−0.0051 (16)	−0.0003 (17)	0.0217 (16)
C8	0.073 (2)	0.072 (2)	0.080 (2)	−0.0082 (18)	−0.021 (2)	−0.0065 (18)
C17	0.102 (3)	0.118 (3)	0.084 (3)	−0.020 (3)	−0.024 (2)	−0.003 (2)
C19	0.077 (2)	0.060 (2)	0.102 (3)	−0.0080 (18)	−0.012 (2)	0.0009 (19)
C1	0.078 (2)	0.060 (2)	0.101 (3)	0.0154 (18)	−0.006 (2)	−0.0072 (19)

### Geometric parameters (Å, º)

**Table:**

O1—C15	1.352 (3)	C5—C4	1.381 (4)
O1—H1	0.843 (18)	C5—C6	1.401 (3)
N1—C9	1.302 (3)	C16—C17	1.507 (4)
N1—C5	1.418 (3)	C16—H16A	0.9700
O3—C2	1.371 (3)	C16—H16B	0.9700
O3—C1	1.420 (3)	C2—C3	1.376 (4)
C13—N2	1.372 (3)	C4—C3	1.390 (3)
C13—C14	1.403 (3)	C4—H4	0.9300
C13—C12	1.423 (3)	C3—H3	0.9300
C14—C15	1.383 (3)	C18—C19	1.511 (4)
C14—H14	0.9300	C18—H18A	0.9700
O2—C6	1.365 (3)	C18—H18B	0.9700
O2—C8	1.424 (3)	C8—H8A	0.9600
C12—C11	1.362 (3)	C8—H8B	0.9600
C12—H12	0.9300	C8—H8C	0.9600
C10—C11	1.410 (3)	C17—H17A	0.9600
C10—C15	1.410 (3)	C17—H17B	0.9600
C10—C9	1.430 (3)	C17—H17C	0.9600
N2—C18	1.456 (3)	C19—H19A	0.9600
N2—C16	1.465 (3)	C19—H19B	0.9600
C11—H11	0.9300	C19—H19C	0.9600
C9—H9	0.9300	C1—H1A	0.9600
C7—C6	1.384 (3)	C1—H1B	0.9600
C7—C2	1.389 (4)	C1—H1C	0.9600
C7—H7	0.9300		
			
C15—O1—H1	105 (3)	O3—C2—C7	114.9 (2)
C9—N1—C5	121.7 (2)	C3—C2—C7	120.5 (3)
C2—O3—C1	117.1 (2)	C5—C4—C3	122.3 (3)
N2—C13—C14	121.2 (2)	C5—C4—H4	118.8
N2—C13—C12	121.5 (2)	C3—C4—H4	118.8
C14—C13—C12	117.3 (2)	O2—C6—C7	124.2 (2)
C15—C14—C13	121.5 (2)	O2—C6—C5	115.8 (2)
C15—C14—H14	119.2	C7—C6—C5	119.9 (2)
C13—C14—H14	119.2	C2—C3—C4	118.6 (3)
C6—O2—C8	117.7 (2)	C2—C3—H3	120.7
C11—C12—C13	121.0 (2)	C4—C3—H3	120.7
C11—C12—H12	119.5	N2—C18—C19	113.1 (3)
C13—C12—H12	119.5	N2—C18—H18A	109.0
C11—C10—C15	117.1 (2)	C19—C18—H18A	109.0
C11—C10—C9	121.2 (2)	N2—C18—H18B	109.0
C15—C10—C9	121.6 (2)	C19—C18—H18B	109.0
C13—N2—C18	122.2 (2)	H18A—C18—H18B	107.8
C13—N2—C16	121.9 (2)	O2—C8—H8A	109.5
C18—N2—C16	115.8 (2)	O2—C8—H8B	109.5
O1—C15—C14	118.2 (2)	H8A—C8—H8B	109.5
O1—C15—C10	120.8 (2)	O2—C8—H8C	109.5
C14—C15—C10	121.0 (2)	H8A—C8—H8C	109.5
C12—C11—C10	122.1 (3)	H8B—C8—H8C	109.5
C12—C11—H11	119.0	C16—C17—H17A	109.5
C10—C11—H11	119.0	C16—C17—H17B	109.5
N1—C9—C10	121.6 (3)	H17A—C17—H17B	109.5
N1—C9—H9	119.2	C16—C17—H17C	109.5
C10—C9—H9	119.2	H17A—C17—H17C	109.5
C6—C7—C2	120.4 (3)	H17B—C17—H17C	109.5
C6—C7—H7	119.8	C18—C19—H19A	109.5
C2—C7—H7	119.8	C18—C19—H19B	109.5
C4—C5—C6	118.3 (2)	H19A—C19—H19B	109.5
C4—C5—N1	123.2 (2)	C18—C19—H19C	109.5
C6—C5—N1	118.3 (2)	H19A—C19—H19C	109.5
N2—C16—C17	112.9 (3)	H19B—C19—H19C	109.5
N2—C16—H16A	109.0	O3—C1—H1A	109.5
C17—C16—H16A	109.0	O3—C1—H1B	109.5
N2—C16—H16B	109.0	H1A—C1—H1B	109.5
C17—C16—H16B	109.0	O3—C1—H1C	109.5
H16A—C16—H16B	107.8	H1A—C1—H1C	109.5
O3—C2—C3	124.6 (3)	H1B—C1—H1C	109.5
			
N2—C13—C14—C15	179.7 (3)	C13—N2—C16—C17	−95.4 (3)
C12—C13—C14—C15	−0.4 (4)	C18—N2—C16—C17	88.7 (3)
N2—C13—C12—C11	−179.3 (3)	C1—O3—C2—C3	−5.4 (5)
C14—C13—C12—C11	0.8 (4)	C1—O3—C2—C7	175.1 (3)
C14—C13—N2—C18	5.4 (4)	C6—C7—C2—O3	179.7 (3)
C12—C13—N2—C18	−174.5 (3)	C6—C7—C2—C3	0.1 (5)
C14—C13—N2—C16	−170.3 (3)	C6—C5—C4—C3	1.3 (4)
C12—C13—N2—C16	9.8 (4)	N1—C5—C4—C3	176.3 (3)
C13—C14—C15—O1	−179.5 (2)	C8—O2—C6—C7	1.1 (4)
C13—C14—C15—C10	0.4 (4)	C8—O2—C6—C5	178.9 (3)
C11—C10—C15—O1	179.1 (3)	C2—C7—C6—O2	177.3 (3)
C9—C10—C15—O1	−4.5 (4)	C2—C7—C6—C5	−0.4 (5)
C11—C10—C15—C14	−0.8 (4)	C4—C5—C6—O2	−178.1 (3)
C9—C10—C15—C14	175.6 (3)	N1—C5—C6—O2	6.6 (4)
C13—C12—C11—C10	−1.2 (4)	C4—C5—C6—C7	−0.2 (4)
C15—C10—C11—C12	1.2 (4)	N1—C5—C6—C7	−175.5 (3)
C9—C10—C11—C12	−175.2 (3)	O3—C2—C3—C4	−178.6 (3)
C5—N1—C9—C10	−169.8 (2)	C7—C2—C3—C4	0.9 (5)
C11—C10—C9—N1	176.0 (3)	C5—C4—C3—C2	−1.6 (5)
C15—C10—C9—N1	−0.2 (4)	C13—N2—C18—C19	−86.8 (3)
C9—N1—C5—C4	33.2 (4)	C16—N2—C18—C19	89.1 (3)
C9—N1—C5—C6	−151.9 (3)		

### Hydrogen-bond geometry (Å, º)

**Table:**

*D*—H···*A*	*D*—H	H···*A*	*D*···*A*	*D*—H···*A*
O1—H1···N1	0.84 (2)	1.79 (2)	2.575 (3)	153 (4)
C16—H16*B*···O1^i^	0.97	2.53	3.491 (4)	172

Symmetry code: (i) *x*−1, *y*, *z*.
